# P-406. Drug Use Evaluation of Ceftriaxone in Pediatrics at a Tertiary Care Hospital in the UAE

**DOI:** 10.1093/ofid/ofaf695.623

**Published:** 2026-01-11

**Authors:** Haya Hamd Al Ali, Waeil Al Naeem

**Affiliations:** Sheikh Khalifa Medical City, Abu Dhabi, Abu Dhabi, United Arab Emirates; Sheikh Khalifa Medical City, Abu Dhabi, Abu Dhabi, United Arab Emirates

## Abstract

**Background:**

Ceftriaxone is a third-generation cephalosporin widely used in pediatric settings due to its broad-spectrum activity and low toxicity. However, inappropriate use can lead to antimicrobial resistance, treatment failure, and increased healthcare costs. This Drug Use Evaluation (DUE) aims to assess the appropriateness of ceftriaxone prescribing among hospitalized pediatric patients at Sheikh Khalifa Medical City (SKMC) based on established clinical guidelines and stewardship principles.

**Table 1.**
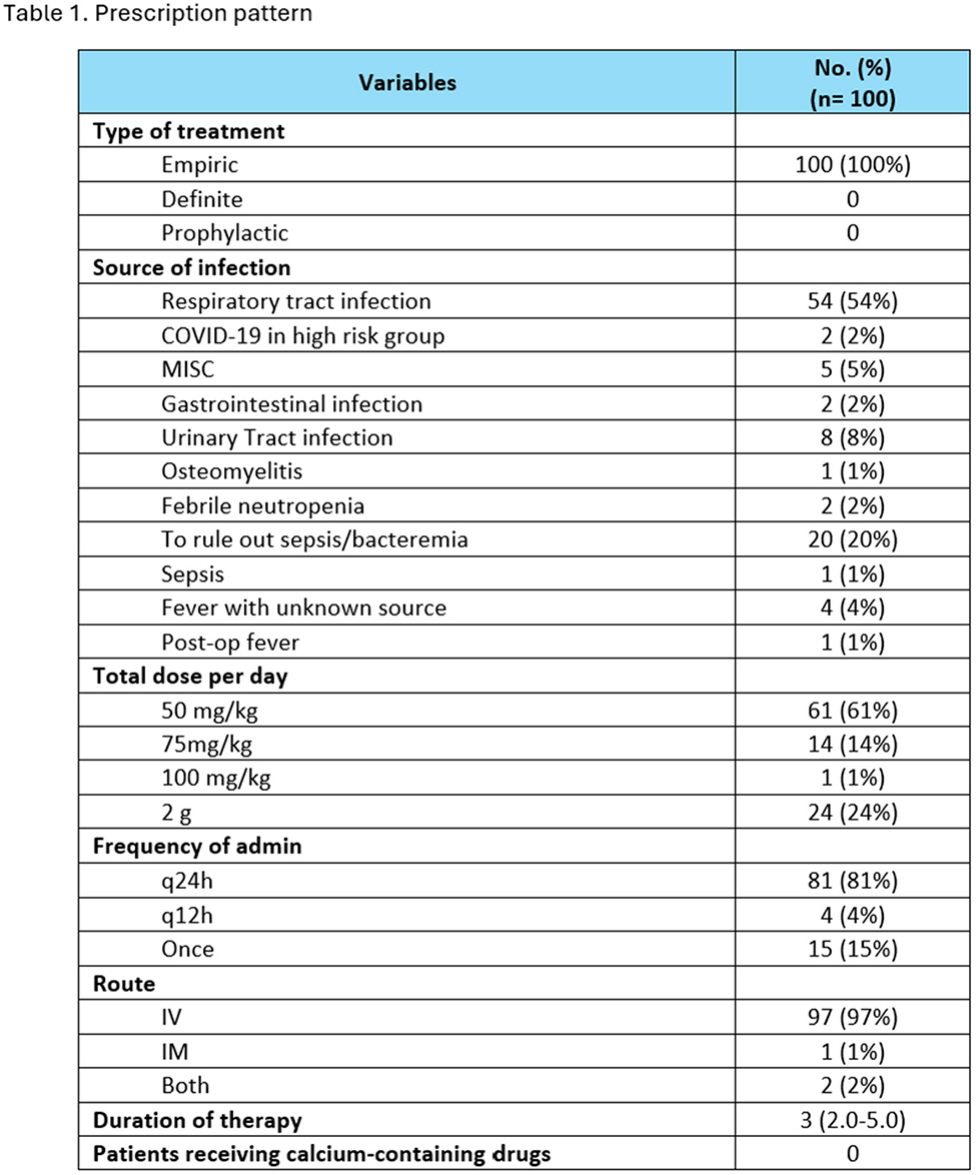
Prescription pattern

**Figure 1.**
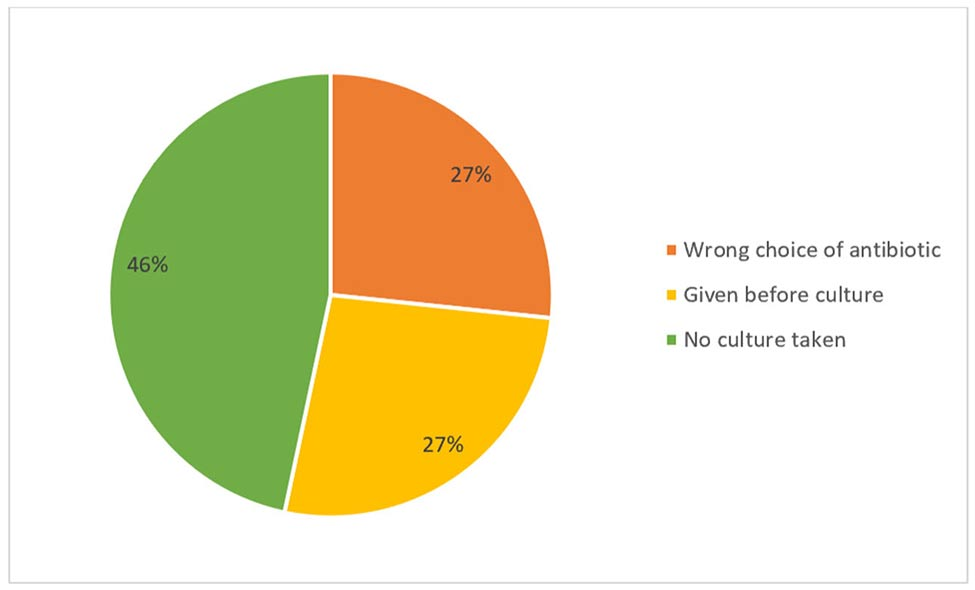
Reasons for inappropriate use

**Methods:**

A retrospective observational study was conducted on 100 pediatric patients prescribed ceftriaxone between June 1 and July 31, 2021, at SKMC. Medical records were reviewed to assess prescription appropriateness based on six parameters: indication, dose, frequency, duration, culture and sensitivity testing, and potential drug interactions. Ceftriaxone use was considered appropriate if all criteria were met. Data sources included PubMed, UpToDate, Lexicomp, and clinical guidelines from the Infectious Diseases Society of America (IDSA), among others.

**Results:**

Among the 100 pediatric patients evaluated, ceftriaxone was prescribed empirically in all cases, most commonly for lower respiratory tract infections (54%), followed by suspected sepsis or bacteremia (20%). Cultures were obtained in 92% of patients, with 94.6% collected before antibiotic administration. However, 15% of cases were deemed inappropriate, primarily due to failure to obtain cultures (46.7%), delayed culture collection (26.6%), and inappropriate antibiotic selection (26.6%). Inappropriate antibiotic choices included use of ceftriaxone in patients with infections requiring broader coverage or alternative agents based on guidelines, such as febrile neutropenia or Enterococcus faecalis urinary tract infections. Overall, 85% of cases met all appropriateness criteria based on international clinical guidelines.

**Conclusion:**

In conclusion, ceftriaxone was appropriately prescribed in the majority of cases, but delays in culture collection and inappropriate antibiotic choices suggest opportunities for improvement. Timely diagnostics and adherence to antimicrobial stewardship principles are essential for optimizing antibiotic use, reducing resistance, and improving patient outcomes.

**Disclosures:**

All Authors: No reported disclosures

